# Prevalence and risk factors of chronic kidney disease and diabetic kidney disease in a central Chinese urban population: a cross-sectional survey

**DOI:** 10.1186/s12882-020-01761-5

**Published:** 2020-04-03

**Authors:** Jia-Yu Duan, Guang-Cai Duan, Chong-Jian Wang, Dong-Wei Liu, Ying-Jin Qiao, Shao-Kang Pan, Deng-Ke Jiang, Yong Liu, Zi-Hao Zhao, Lu-Lu Liang, Fei Tian, Zhang-Suo Liu

**Affiliations:** 1 Department of Nephrology, The First Affiliated Hospital of Zhengzhou University, Research Institute of Nephrology, Zhengzhou University, Jianshe Road No.1, Zhengzhou, 450052 Henan People’s Republic of China; 2 Department of Epidemiology and Biostatistics, College of Public Health, Zhengzhou University, Zhengzhou, Henan People’s Republic of China

**Keywords:** Prevalence, Ordinal logistic regression, Chronic kidney disease, Diabetic kidney disease

## Abstract

**Background:**

This study was conducted to evaluate and update the current prevalence of and risk factors for chronic kidney disease (CKD) and diabetic kidney disease (DKD) in a central Chinese urban population.

**Methods:**

From December 2017 to June 2018, a total of 5231 subjects were randomly enrolled from 3 communities in 3 districts of Zhengzhou. CKD was defined as estimated glomerular filtration rate (eGFR) < 60 mL/min.1.73m^2^ or urinary albumin to creatinine ratio ≥ 30 mg/g (albuminuria). Diabetic subjects with systolic blood pressure > 140 mmHg, albuminuria or an eGFR less than 60 mL/min/1.73 m^2^ were classified as having DKD. Participants completed a questionnaire assessing lifestyle and relevant medical history, and blood and urine specimens were taken. Serum creatinine, uric acid, total cholesterol, triglycerides, low-density lipoprotein, high-density lipoprotein and urinary albumin were assessed. The age- and sex-adjusted prevalences of CKD and DKD were calculated, and risk factors associated with the presence of reduced eGFR, albuminuria, DKD, severity of albuminuria and progression of reduced renal function were analyzed by binary and ordinal logistic regression.

**Results:**

The overall adjusted prevalence of CKD was 16.8% (15.8–17.8%) and that of DKD was 3.5% (3.0–4.0%). Decreased renal function was detected in 132 participants (2.9, 95% confidence interval [CI]: 2.5–3.2%), whereas albuminuria was found in 858 participants (14.9, 95% CI: 13.9–15.9%). In all participants with diabetes, the prevalence of reduced eGFR was 6.3% (95% CI = 3.9–8.6%) and that of albuminuria was 45.3% (95% CI = 40.4–50.1%). The overall prevalence of CKD in participants with diabetes was 48.0% (95% CI = 43.1–52.9%). The results of the binary and ordinal logistic regression indicated that the factors independently associated with a higher risk of reduced eGFR and albuminuria were older age, sex, smoking, alcohol consumption, overweight, obesity, diabetes, hypertension, dyslipidemia and hyperuricemia.

**Conclusions:**

Our study shows the current prevalence of CKD and DKD in residents of Central China. The high prevalence suggests an urgent need to implement interventions to relieve the high burden of CKD and DKD in China.

## Background

The prevalences of chronic kidney disease (CKD) and diabetic kidney disease (DKD) significantly increased in the past decades worldwide. Both CKD and DKD have been a global health issue because of the anonymous symptoms, insufficient awareness and rapid progression in their late stages. The results of global burden of disease study in 2017 showed that the incidence, prevalence and mortality of CKD has increased 31.6, 27.0 and 34.0% comparing with which in 2007, respectively [1–4].

In the past decade, CKD has been highly prevalent in China. According to the Chinese National Renal Data System, there were a total of 339,748 maintenance hemodialysis patients in China by 2014 [5]. The elevated prevalence of relevant risk factors, such as diabetes, hypertension, hyperuricemia and dyslipidemia play an important role [6–9]. In 2010, Yang et al. reported that the prevalences of diabetes and prediabetes were 9.7 and 15.5%, respectively, in China [10]. In the same year, Ho and Hwang et al. demonstrated that diabetic kidney disease (DKD) was responsible for 46.2 and 43.2% of ESRD cases in Hong Kong and Taiwan, respectively [11, 12]. The inadequate awareness and control of diabetes and hypertension has aggravated the health and socioeconomic burden of CKD and DKD in the Chinese population in several aspects, such as lower life expectancy, poor quality of daily life and the high cost of medical care [13–16]. Insufficient contracts with health services, delayed health-seeking behavior and frequent use of Chinese herbal medicines have also contributed to the high incidence and progression of CKD [17, 18].

Previous studies have indicated that over 60% of CKD cases could be detected early by general screening [19–21]. Timely medical care is beneficial for improving the quality of life of CKD patients and reducing the morbidity and mortality caused by ESRD [21]. Nevertheless, studies reporting epidemiological features of CKD and DKD in the Chinese population are still insufficient. Efforts to update the epidemiological data and identify the early risk factors are urgently needed and will be beneficial for developing effective strategies for the prevention of CKD, DKD and ESRD. Therefore, we conducted a cross-sectional study to provide current epidemiological data on CKD and DKD and to identify their risk factors in a Central Chinese urban population.

## Methods

### Study subjects

From December 2017 to June 2018, the subjects were recruited from 3 communities in 3 districts of Zhengzhou: the Erqi, Zhongyuan and Jinshui districts. There are 12 administrative districts with a total population of approximately 10 million in Zhengzhou (data available on http://tjj.zhengzhou.gov.cn/). A multistage, stratified cluster sampling method was employed to select participants over 18 years old from the general population. In the first stage, 3 districts were randomly selected from 6 urban districts. In the second stage, one representative community in each district was selected according to the proportion of permanent residents. In the final stage, all permanent residents who satisfied the inclusion criteria and agreed to sign the informed consent were recruited in this study. Altogether, a total of 6000 subjects aged 18 years or older were selected from 3 communities, and 5231 subjects completed the survey and examination, corresponding to a response rate of 87.2%. Two hundred and thirty-three subjects were dropped because they didn’t respond and 536 subjects were dropped because they didn’t complete the questionnaire.

### Measurements and definitions

Data were collected by face-to-face interviews in examination centers at community health stations. All subjects completed a questionnaire that collected information about their sociodemographic status, personal and family health history, lifestyle behaviors and awareness and control of chronic noncommunicable disease with assistance of trained practitioners and medical employees. Anthropometric measurements, such as height, weight and blood pressure (BP), were obtained. Subjects were required to keep light clothing without shoes when measuring the height and weight. BMI was divided into four levels: underweight (< 18.5 kg/m^2^), healthy weight (18.5–23.9 kg/m^2^), overweight (24–27.9 kg/m^2^) and obesity (≥ 28 kg/m^2^) by using the Chinese “Criteria of weight for adults (No. WS/T 428-2013)”. BP was measured using an electronic sphygmomanometer (Omron HEM-7071A, Japan) three times with an one-minute interval. The mean of the three BP values was used for statistical analysis unless the interpolation between the values was higher than 10 mmHg, in which case the mean of the other two closest results was utilized. Hypertension was defined as an average systolic BP (SBP) ≥ 140 mmHg and/or an average diastolic BP (DBP) ≥ 90 mmHg or the participant was diagnosed hypertension and need to take medications to control BP [22]. Subjects with hypertension were considered to have controlled BP if SBP < 140 mmHg and DBP < 90 mmHg.

Participants were required an overnight (at least 8 h) fasting and their venous blood specimens were collected in vacuum tubes without an anticoagulant. Serum concentrations of creatinine, uric acid, total cholesterol, triglycerides, high-density lipoprotein, low-density lipoprotein and fasting plasma glucose (FPG) level were measured. Urinary albumin and creatinine were measured from a fresh morning spot urine sample and the urinary albumin to creatinine ratio (ACR, mg/g) was calculated substantially.

The estimated glomerular filtration rate (eGFR) was calculated by serum creatinine using the 2009 CKD-EPI creatinine equation [23]. According to the 2012 KDIGO classification, eGFR was classified into 5 stages, in which stage 3 was further divided into stages 3a and 3b with an eGFR of 45 mL/min/1.73 m^2^ as the cut-off value. ACR was classified as follows: A1, < 30 mg/g; A2, 30–300 mg/g; and A3, > 300 mg/g [24]. Albuminuria was defined as an ACR ≥ 30 mg/g. Indicators of renal damage were the presence of an eGFR less than 60 mL/min/1.73 m^2^ or albuminuria. CKD was defined as the presence of one or two indicators of renal damage.

Diabetes was defined as: 1. FPG ≥ 7.0 mmol/L; 2. using insulin or taking antidiabetic medications to control serum glucose in the past 2 weeks; and 3. being diagnosed diabetes by a physician. Diabetic subjects with systolic blood pressure > 140 mmHg, albuminuria or an eGFR less than 60 mL/min/1.73 m^2^ were classified as having DKD. Control of diabetes was defined as FPG maintained at less than 7.0 mmol/L for the past 7 days. Dyslipidemia was considered the use of anti-dyslipidemia medications during the last 2 weeks or the presence of one or more abnormal serum lipid concentrations according to the Chinese guidelines for the prevention and treatment of dyslipidemia in adults: 1. Total cholesterol > 6.22 mmol/L; 2. Triglycerides > 2.26 mmol/L; 3. High-density lipoprotein cholesterol < 1.04 mmol/L; 4. Low-density lipoprotein cholesterol > 4.14 mmol/L [25]. Hyperuricemia was defined as a serum uric acid concentration higher than 422 μmol/L (4.77 mg/dL) for men and 363 μmol/L (4.10 mg/dL) for women.

Education was classified into 3 categories: 1. ≤ Primary school; 2. junior middle school; and 3. ≥ senior high school. Diet rich in fruits and vegetables Diet with a daily average consumption of more than 500 g of fruits and vegetables was classified as a diet rich in fruits and vegetables. A high-fat diet was confirmed when participant consuming livestock and poultry over 75 g per day [26]. Physical activity was categorized into three levels by using the international physical activity questionnaire (IPAQ 2001) [27].

### Statistical analysis

Epidata software (version 3.1) was employed for data entry and management. All statistical analyses were performed on SAS 9.1 (SAS Institute, Cary, NC, USA) and GraphPad Prism 6 (GraphPad Software, Inc., La Jolla, CA, USA) for Windows. A *P* <  0.05 was considered statistically significant. Data are expressed as the mean ± SD or median with range for continuous variables and frequency (percentage) for discrete variables, as appropriate. Intergroup comparisons were performed using The Pearson *chi-square* test, Student’s t-test, Mann-Whitney U-test and Wilcoxon test were employed to verify the statistical significance of intergroup comparison. The standard population of this study was based on data from the China Population Sampling Census in 2009 (data available on http://www.stats.gov.cn/).

The crude and adjusted prevalences of reduced eGFR (eGFR < 60 mL/min/1.73 m^2^), albuminuria, DKD and CKD were calculated. Both binary and ordinal logistic regressions were employed to explore the associations between indicators of renal damage and the relevant covariates. In binary logistic regression, crude and multivariable adjusted odds ratios (ORs) with 95% confidential intervals (CIs) were calculated. The reference values of covariates in our multivariable logistic regression model were: age, 18–29 years old (each unit increase refers to 10 years older); gender, women women; education, ≤ primary school; current smoker, no; alcohol consumption, no; BMI, healthy weight; diabetes, no; hypertension, no; dyslipidemia, no; hyperuricemia, no. According to the prognosis of CKD by GFR and albuminuria category from the 2012 Kidney Disease: Improving Global Outcomes (KDIGO) guidelines, two models were used to calculate the data in the ordinal logistic regression. In model 1, we analyzed data from subjects with an eGFR > 60 mL/min/1.73 m^2^ and different levels of ACR (A1 – A3, *n* = 5099) [24]. In model 2, data from all participants were divided into 4 groups as follows: low risk (no CKD); moderately increased risk; high risk; and very high risk [24]. The results of tests of parallel lines indicated that the two models were statistically executable (both *P* values > 0.05).

## Results

Of the 6000 participants involved in this study, 5231 had a complete data set and were entered into our statistical analysis. Their demographic and clinical characteristics are shown in Table 1. The prevalences of hypertension, dyslipidemia, hyperuricemia and diabetes were 34.6, 14.6, 11.5 and 7.6%, respectively. A total of 80.3% of the participants attended senior high school. The prevalences of current smokers and habitual drinkers were similar. The mean eGFR was 92.6 ± 21.5 mL/min/1.73 m^2^, and the median ACR was 14.1, with an interquartile range of 8.8 to 23 mg/g. Generally, participants with reduced eGFR or albuminuria were older, primary educated, and performed heavy physical activities. They had a lower proportion of high fat diet and higher prevalences of cardiovascular disease, hypertension, dyslipidemia, hyperuricemia and diabetes than did those without indicators of renal damage.

**Table 1:** General characteristics of participants according to indicators of renal damage

	Participants with eGFR < 60 mL/min/1.73 m^2^ (*N* = 132)	Participants with albuminuria (*N* = 859)	Participants with DKD (*N* = 92)	Participants without renal damage (*N* = 4286)	Total (*N* = 5231)
Age	63.0 (13.8)	51.5 (14.0)	60.8 (11.8)	40.3 (16.2)	42.5 (16.5)
Men	24 (18.2%)	574 (66.8%)	62 (67.4%)	2362 (55.1%)	2945 (56.3%)
Education					
≤ Primary school	20 (15.2%)	90 (10.5%)	15 (16.3%)	156 (3.6%)	257 (4.9%)
Junior high school	32 (24.2%)	192 (22.4%)	26 (28.3%)	564 (13.2%)	774 (14.8%)
≥ Senior high school	80 (60.6%)	577 (67.2%)	51 (55.4%)	3566 (83.2%)	4200 (80.3%)
Current smoker	9 (6.8%)	295 (34.3%)	29 (31.5%)	825 (19.2%)	1125 (21.6%)
Habitual drinker	7 (5.3%)	282 (32.8%)	32 (34.8%)	817 (19.1%)	1104 (21.1%)
Dietary pattern					
Diet rich in fruits and vegetables	78 (59.1%)	419 (48.8%)	38 (41.3%)	1746 (40.7%)	2222 (42.5%)
High fat diet	11 (8.3%)	92 (10.7%)	9 (9.8%)	680 (15.9%)	779 (14.9%)
Physical activity					
Low	99 (75%)	494 (57.5%)	55 (59.8%)	2911 (67.9%)	3469 (66.3%)
Moderate	33 (25%)	330 (38.4%)	31 (33.7%)	1293 (30.2%)	1645 (31.4%)
High	0 (0%)	35 (4.1%)	6 (6.5%)	83 (1.9%)	117 (2.2%)
Self-reported HBV infection	2 (1.5%)	11 (1.3%)	0 (0.0%)	54 (1.3%)	66 (1.3%)
Cardiovascular disease	37 (28%)	112 (13%)	29 (31.5%)	247 (5.8%)	376 (7.2%)
Hypertension	79 (59.8%)	558 (65%)	NA	1213 (28.3%)	1812 (34.6%)
Dyslipidemia	33 (25%)	248 (28.9%)	37 (40.2%)	496 (11.6%)	765 (14.6%)
Hyperuricemia	20 (15.2%)	142 (16.5%)	12 (13.0%)	447 (10.4%)	599 (11.5%)
Diabetes	25 (18.9%)	181 (21.1%)	NA	208 (4.9%)	400 (7.6%)
Body mass index (kg/m^2^)	24.9 (3.6)	26.1 (3.8)	26.5 (3.2)	23.6 (3.6)	24.1 (3.8)
Waist–hip ratio	0.8 (0.05)	0.9 (0.04)	0.9 (0.03)	0.8 (0.1)	0.8 (0.1)
Total cholesterol (mmol/L)	4.8 (0.9)	4.6 (0.97)	4.7 (0.9)	4.2 (0.9)	4.3 (0.9)
Triglyceride (mmol/L)	1.7 (1.2)	2.0 (1.8)	2.4 (2.0)	1.3 (1.1)	1.4 (1.3)
LDL cholesterol (mmol/L)	2.6 (0.7)	2.5 (0.6)	2.6 (0.6)	2.3 (0.7)	2.3 (0.7)
HDL cholesterol (mmol/L)	1.4 (0.3)	1.3 (0.4)	1.2 (0.3)	1.4 (0.4)	1.3 (0.4)
Fasting blood glucose (mmol/L)	5.4 (2.3)	5.8 (2.2)	9.1 (3.0)	4.9 (1.0)	5.0 (1.3)
Uric acid (μmol/L)	285.5 (94.5)	321.7 (102.1)	302.4 (99.4)	289.7 (95.5)	294.6 (97.3)
Creatinine (μmol/L)	97.8 (92.0–106.7)	84.0 (39.5)	82.3 (70.4–96.9)	79.6 (15.2)	80.8 (25.1)
eGFR (mL/min/1.73m^2^)	52.4 (10.7)	86.9 (19.4)	79.6 (21.7)	94.5 (21.2)	92.6 (21.5)
ACR (mg/g)	20.1 (10.7–44.2)	47.8 (36.6–106.3)	73.9 (42.4–244.8)	12.3 (8.1–17.6)	14.1 (8.8–23)

Note: Data were n (%), mean (standard deviation) or median with interquartile range, as appropriate

Abbreviations: *DKD* Diabetic kidney disease, *HBV* Hepatitis B virus, *LDL* Low density lipoprotein, *HDL* High density lipoprotein, *eGFR* Estimated glomerular filtration rate, *ACR* Albumin: creatinine ratio, *NA* Not applicable

**Fig. 1:**
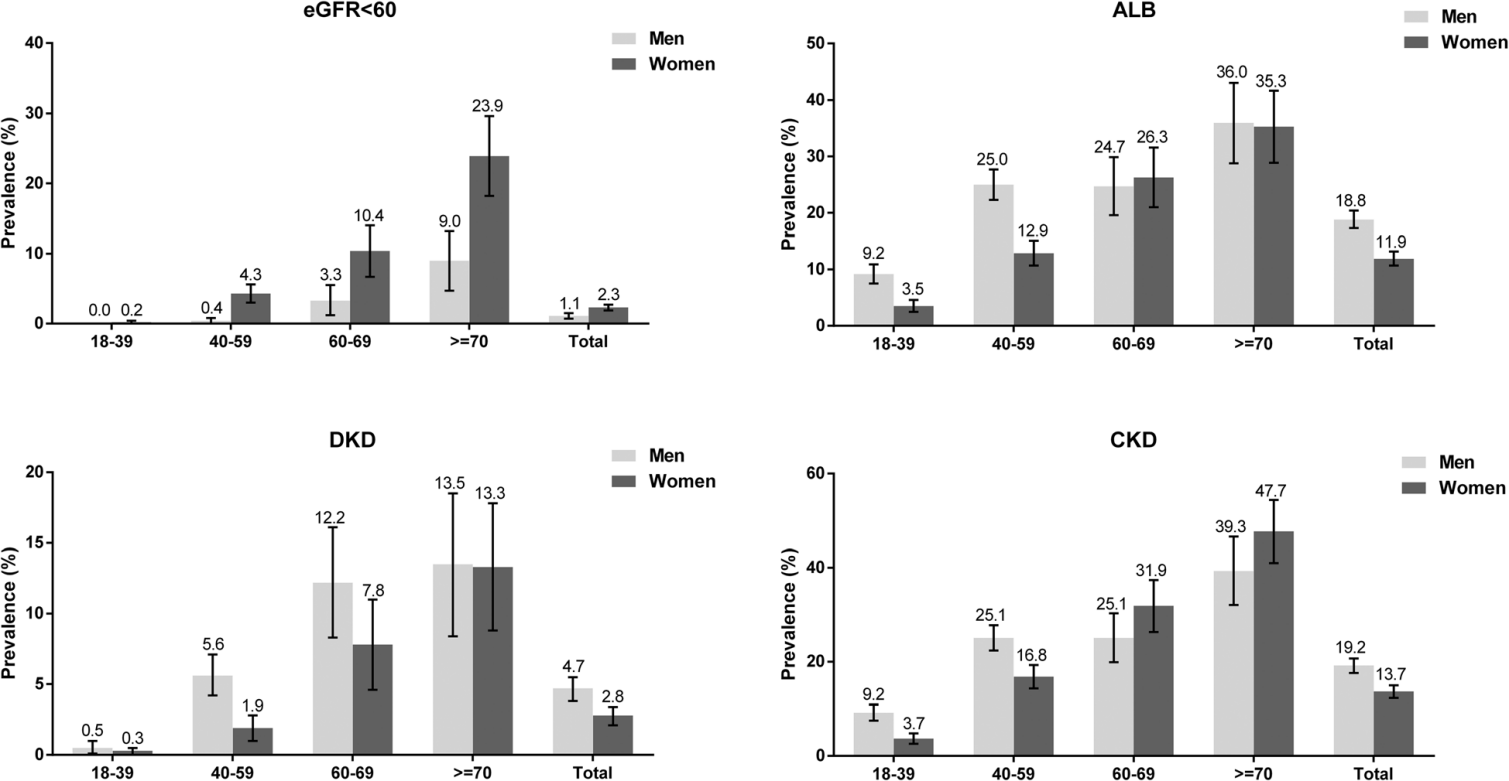
Adjusted prevalence of indicators of renal damage, DKD and CKD, stratified by sex and age. Adjusted prevalences of eGFR less than 60 mL/min/1.73 m^2^ (eGFR < 60), albuminuria (ALB), diabetic kidney disease (DKD) and chronic kidney disease (CKD). Data are expressed as prevalence, and the bars represent the 95% CIs

**Fig. 2:**
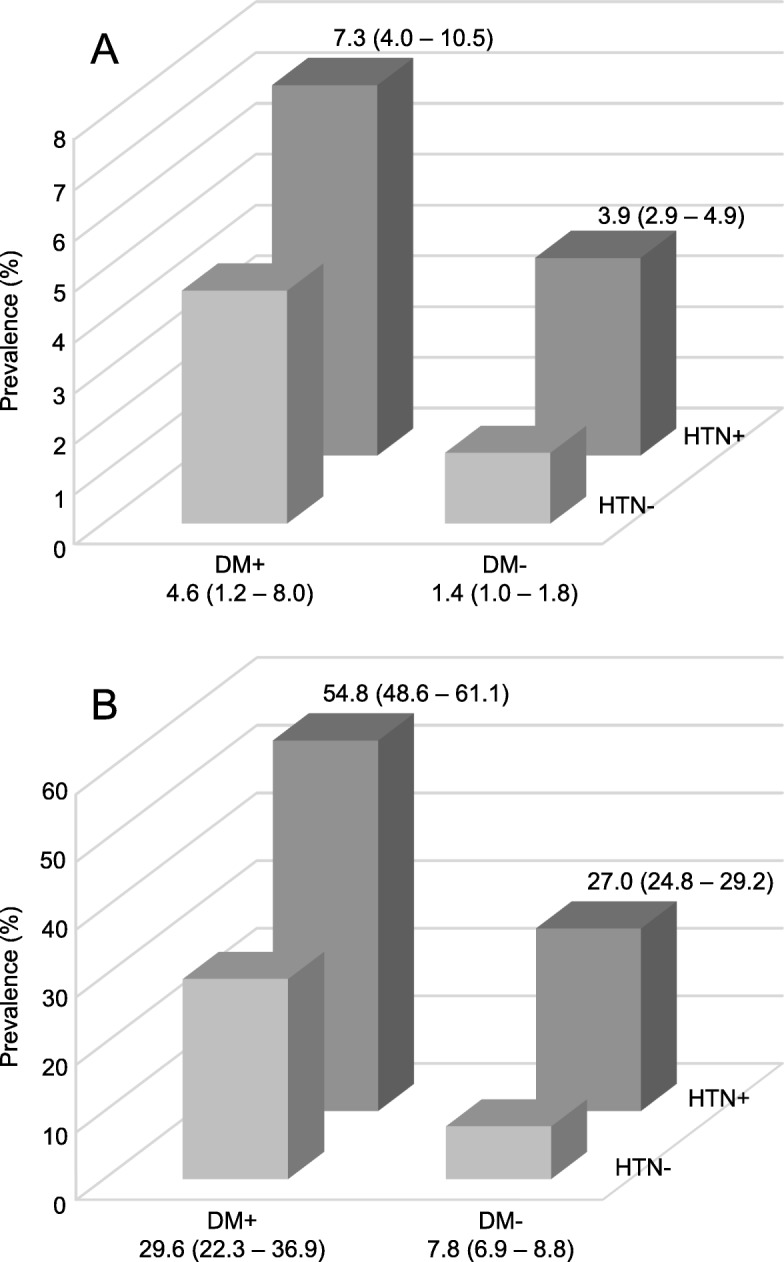
Prevalence of indicators of renal damage according to hypertension and diabetes. Prevalences of eGFR less than 60 mL/min/1.73 m^2^ (**a**) and albuminuria (**b**). Data are expressed as prevalence (95% CI for prevalence). DM refers to diabetes mellitus, and HTN refers to hypertension

There were 132 subjects exhibiting an eGFR less than 60 mL/min/1.73 m^2^ and 858 subjects exhibiting albuminuria (Table 2). A total of 945 subjects had CKD and 92 of them were DKD patients. The adjusted prevalence of reduced eGFR was 2.8% (95% CI = 2.4–3.3%) and that of albuminuria was 14.9% (95% CI = 13.9–15.9%). The overall adjusted prevalences of CKD and DKD were 16.8% (95% CI = 15.8–17.8%) and 1.8% (95% CI = 1.4–2.1%), respectively. By disease stage, the prevalence was as follows: stage 1, 6.0%; stage 2, 7.8%; stage 3a, 2.4%; stage 3b, 0.2%; stage 4, 0.3% and stage 5, 0.1%. In subjects with normal eGFR, the numbers with stages A1 – A3 were 4286, 743 and 70, respectively. As shown in Fig. 1, the prevalence of reduced eGFR was much higher in women over 40 years old than in their male counterparts, and the overall prevalence was higher with older age in both men and women. The prevalence of albuminuria was higher in older participants and in women than men in all age groups except for women subjects who aged 60–69 years old (24.7 versus 26.3%). Generally, the prevalences of CKD and DKD increased along with age in men and women subjects. In addition, subjects with both hypertension and diabetes shows the highest prevalences of reduced eGFR (7.3 95% CI = 4.0–10.5%) and albuminuria (54.8 95% CI = 48.6–61.1%) than whom with either hypertension or diabetes (Fig. 2).

**Table 2:** Adjusted prevalence of indicators of renal function and chronic kidney disease, by disease stage

Renal indicator								Chronic kidney disease (in 5231 participants)	
	eGFR (mL/min/1.73m^2^)			Albuminuria		Diabetic kidney disease			
Stage		n	Prevalence (95% CI)	n	Prevalence (95% CI)	n	Prevalence (95% CI)	N	Prevalence (95% CI)
1	> 90	2744	52.1 (50.7–53.4)	370	11.5 (10.3–12.7)	30	1.1 (0.7–1.5)	370	6.0 (5.3–6.6)
2	60–89	2355	42.4 (41.1–43.8)	443	18.4 (16.8–20.0)	49	2.1 (1.5–2.7)	443	7.8 (7.1–8.5)
3	30–59	124	2.6 (2.2–3.1)	40	37.0 (28.8–45.1)	10	8.1 (3.2–12.9)	124	2.6 (2.2–3.1)
3a	45–59	115	2.4 (2.0–2.8)	33	32.8 (24.5–41.1)	9	7.8 (2.8–12.8)	115	2.4 (2.0–2.8)
3b	30–44	9	0.2 (0.1–0.4)	7	76.9 (50.4–93.4)	1	11.1 (1.45–36.7)	9	0.2 (0.1–0.4)
4	15–29	5	0.2 (0.1–0.4)	4	46.2 (14.8–77.5)	2	40.0 (2.8–70.8)	5	0.2 (0.1–0.4)
5	< 15	3	0.1 (0.1–0.2)	1	14.3 (9.4–29.2)	1	14.3 (9.4–29.2)	3	0.1 (0.1–0.2)
Total		5231	100	858	14.9 (13.9–15.9)	92	1.8 (1.4–2.1)	945	16.8 (15.8–17.8)

Note: Albuminuria was defined as urinary albumin to creatinine ratio ≥ 30 mg/g creatinine. CKD was defined as an eGFR less than 60 mL/min/1.73m^2^ or presence of albuminuria. All prevalences were adjusted for synthesized weights

Abbreviations: *eGFR* Estimated glomerular filtration rate

Comparing with those who have no renal damages, subjects with reduced eGFR performed an older age, more women individuals, insufficient consumption of meat and physical activity, poor control of diabetes and more hypertension and dyslipidemia, while those with albuminuria tended to exhibit poor control of diabetes and more dyslipidemia (Table 3). Twenty-five subjects were classified as stage 3–5 CKD, and 181 subjects had albuminuria (Table 4). The prevalence of reduced eGFR was 6.3% (95% CI = 3.9–8.6%), and that of albuminuria was 45.3% (95% CI = 40.4–50.1%). The overall prevalence of CKD in diabetic subjects was 48.0% (95% CI = 43.1–52.9%). By disease stage, the prevalence was as follows: stage 1, 15.8%; stage 2, 26.0%; stage 3a, 2.0%; stage 3b, 0.8%; stage 4, 0.5% and stage 5, 0.3%. In subjects with normal eGFR, the numbers with stages A1 – A3 were 209, 139 and 28, respectively.

**Table 3:** General characteristics of participants with diabetes according to indicators of renal damage

	Participants with eGFR < 60 mL/min/1.73 m^2^ (*N* = 25)	Participants with albuminuria (*N* = 181)	Participants without renal damage (*N* = 208)	Total (*N* = 400)
Age	68.4 (11.3)	57.6 (12.4)	57.2 (10.6)	57.5 (11.5)
Men	5 (20.0%)	132 (72.9%)	123 (59.1%)	255 (63.8%)
Education				
≤ Primary school	2 (8.0%)	26 (14.4%)	21 (10.1%)	48 (12.0%)
Junior high school	9 (36.0%)	51 (28.2%)	67 (32.2%)	121 (30.3%)
≥ Senior high school	14 (56.0%)	104 (57.5%)	120 (57.7%)	231 (57.8%)
Current smoker	1 (4.0%)	66 (36.5%)	55 (26.4%)	121 (30.3%)
Habitual drinker	1 (4.0%)	61 (33.7%)	35 (16.8%)	96 (24.0%)
Dietary pattern				
Diet rich in fruits and vegetables	9 (36.0%)	69 (38.1%)	100 (48.1%)	174 (43.5%)
High fat diet	1 (4.0%)	16 (8.8%)	22 (10.6%)	38 (9.5%)
Physical activity				
Low	19 (76.0%)	101 (55.8%)	131 (63.0%)	242 (60.5%)
Moderate	6 (24.0%)	72 (39.8%)	74 (35.6%)	147 (36.8%)
High	0 (0.0%)	8 (4.4%)	3 (1.4%)	11 (2.8%)
Awareness of diabetes	24 (96.0%)	130 (71.8%)	164 (78.8%)	305 (76.3%)
Control of diabetes	9 (36.0%)	38 (21.0%)	89 (42.8%)	133 (33.3%)
Self-reported HBV infection	0 (0.0%)	3 (1.7%)	11 (5.3%)	14 (3.5%)
Hypertension	18 (72.0%)	136 (75.1%)	106 (51.0%)	248 (62.0%)
Dyslipidemia	13 (52.0%)	67 (37.0%)	40 (19.2%)	113 (28.3%)
Hyperuricemia	3 (12.0%)	21 (11.6%)	19 (9.1%)	40 (10.0%)
Body mass index (kg/m^2^)	26.2 (3.0)	26.7 (3.3)	25.5 (3.4)	26.0 (3.4)
Total cholesterol (mmol/L)	4.7 (0.9)	4.6 (1.0)	4.4 (1.0)	4.5 (1.0)
Triglyceride (mmol/L)	2.2 (1.2)	2.6 (2.5)	1.3 (0.9–2.0)	2.2 (2.3)
LDL cholesterol (mmol/L)	2.5 (0.6)	2.6 (0.7)	2.4 (0.7)	2.5 (0.7)
HDL cholesterol (mmol/L)	1.2 (0.3)	1.2 (0.3)	1.2 (0.3)	1.2 (0.3)
Fasting plasma glucose (mmol/L)	8.6 (3.7)	9.1 (2.9)	7.6 (2.3)	8.3 (2.7)
Uric acid (μmol/L)	282.0 (203.0–347.0)	300.4 (99.1)	272.3 (93.3)	283.6 (97.0)
Creatinine (μmol/L)	97.5 (89.0–111.3)	8.4 (71.6–96.5)	77.8 (17.2)	82.9 (40.0)
eGFR (mL/min/1.73m^2^)	48.8 (12.9)	82.4 (20.1)	85.6 (16.5)	83.3 (18.6)
ACR (mg/g)	34.6 (12.4–172.7)	62.7 (39.4–196.7)	16.3 (7.5)	27.8 (14.5–53.5)

Note: Data were n (%), mean (standard deviation) or median with interquartile range, as appropriate

Abbreviations: *DKD* Diabetic kidney disease, *HBV* Hepatitis B virus, *LDL* Low density lipoprotein, *HDL* High density lipoprotein, *eGFR* Estimated glomerular filtration rate, *ACR* Albumin: creatinine ratio

**Table 4:** Prevalence of indicators of renal damage in participants with diabetes, by disease stage

	eGFR (mL/min/1.73m^2^)			Albuminuria	
Stage		n	Prevalence (95% CI)	n	Prevalence (95% CI)
1	> 90	143	35.8 (31.0–40.5)	63	15.8 (12.2–19.3)
2	60–89	232	58.0 (53.1–62.9)	104	26.0 (21.7–30.3)
3	30–59	22	5.5 (3.3–7.7)	11	2.8 (1.1–4.4)
3a	45–59	19	4.8 (2.7–6.8)	8	2.0 (0.6–3.4)
3b	30–44	3	0.8 (0.1–1.6)	3	0.8 (0.1–1.6)
4	15–29	2	0.5 (0.1–1.2)	2	0.5 (0.1–1.2)
5	< 15	1	0.3 (0.1–0.7)	1	0.3 (0.1–0.7)
Total		400	100	181	45.3 (40.4–50.1)

Note: Albuminuria was defined as urinary albumin to creatinine ratio ≥ 30 mg/g creatinine

Abbreviations: *eGFR* Estimated glomerular filtration rate

The prevalence of reduced eGFR was not significantly different among the three tertiles of education and family income, while that of albuminuria was highest in subjects in the lower tertile of education and upper tertile of family income (Table 5). The overall adjusted prevalences of CKD were 33.5% (95% CI = 27.7–39.3%) and 26.5% (95% CI = 22.4–30.6%), respectively. The prevalences of hypertension and diabetes were lower in subjects with higher education conditions, while they were lowest in subjects in the middle tertile of family income. Poor control of hypertension and diabetes was most prevalent in subjects in the upper tertile of education (6.9 and 29.9%, respectively) and middle tertile of family income (9.3 and 27.4%, respectively).

**Table 5:** Adjusted prevalence of indicators of renal damage, hypertension and diabetes, by education and family income

	eGFR < 60 mL/min/1.73m^2^	Albuminuria	CKD	Hypertension	Control of hypertension	Diabetes	Control of diabetes	DKD
Education								
≤ Primary school, tertile 1	2.3 (0.5–4.2)	31.9 (26.2–37.6)	33.5 (27.7–39.3)	51.8 (45.6–57.9)	15.8 (9.5–22.1)	18.7 (13.9–23.5)	33.3 (19.5–47.2)	5.8 (3.0–8.7)
Junior high school, tertile 2	2.1 (1.1–3.1)	29.6 (26.4–32.8)	31.0 (27.7–34.3)	47.9 (44.4–51.5)	15.6 (11.9–19.3)	15.6 (13.1–18.2)	39.7 (30.8–48.5)	3.4 (2.1–4.6)
≥ Senior high school, tertile 3	3.2 (2.7–3.7)	16.1 (15.0–17.2)	17.9 (16.7–19.0)	31.1 (29.7–32.5)	6.9 (5.5–8.3)	5.5 (4.8–6.2)	29.9 (23.9–35.8)	1.2 (0.9–1.5)
^*^P_trend_	0.20	< 0.001	< 0.001	< 0.001	< 0.001	< 0.001	0.18	< 0.001
Family monthly income (RMB)								
≤ 5000, tertile 1	3.7 (2.0–5.5)	18.2 (14.6–21.7)	20.6 (16.8–24.3)	55.1 (50.6–59.7)	21.3 (16.3–26.4)	19.3 (15.6–22.9)	44.3 (33.7–54.9)	6.8 (4.5–9.1)
5000 –, tertile 2	2.6 (1.9–3.3)	18.1 (16.3–19.9)	20.0 (18.2–21.8)	35.4 (33.2–37.6)	9.3 (7.1–11.6)	6.7 (5.6–7.9)	27.4 (19.5–35.4)	1.0 (0.6–1.5)
≥ 7000, tertile 3	2.4 (1.0–3.9)	25.2 (21.2–29.2)	26.5 (22.4–30.6)	39.3 (34.8–43.8)	12.9 (7.9–17.9)	11.9 (8.9–14.9)	33.3 (20.3–46.3)	2.9 (1.3–4.4)
^*^P_trend_	0.38	0.002	0.01	< 0.001	< 0.001	< 0.001	0.04	< 0.001

Note: Albuminuria was defined as urinary albumin to creatinine ratio ≥ 30 mg/g creatinine. CKD was defined as an eGFR less than 60 mL/min/1.73m^2^ or presence of albuminuria. All prevalences were adjusted for synthesized weights

Abbreviations: *eGFR* Estimated glomerular filtration rate, *CKD* Chronic kidney disease, *DKD* Diabetic kidney disease

^*^P_trend_ was calculated by Cochran-Armitage test

The results of the binary logistic regression are shown in Table 6. Older age, higher education, and hypertension were all independently associated with a higher risk of reduced eGFR, while male gender showed the opposite association. Factors independently associated with a higher risk of albuminuria were older age, being a current smoker, a diet rich in fruits and vegetables, overweight, obesity, diabetes, hypertension and dyslipidemia. Higher education level and more consumption of meat were associated with lower ORs of developing albuminuria than the other factors. In addition, being male and over 40 years of age, high level physical activity, obesity, dyslipidemia and hyperuricemia were significantly associated with an increased risk of DKD.

**Table 6:** Factors associated with indicators of renal damage and diabetic kidney disease

	eGFR < 60 ml/min/1.73m^2^		Albuminuria		Diabetic kidney disease	
Age	Crude OR	Adjusted OR	Crude OR	Adjusted OR	Crude OR	Adjusted OR
18-	1.00	1.00	1.00	1.00	1.00	1.00
Age changed by 10 years	2.71 (2.34–3.13)	2.77 (2.32–3.32)	1.61 (1.52–1.71)	1.18 (1.08–1.28)	2.19 (1.87–2.57)	2.26 (1.85–2.77)
Gender						
Women	1.00	1.00	1.00	1.00	1.00	1.00
Men	0.17 (0.11–0.26)	0.16 (0.09–0.29)	1.70 (1.46–1.98)	1.18 (0.96–1.47)	1.62 (1.04–2.51)	1.85 (1.04–3.28)
Education						
≤ Primary school	1.00	1.00	1.00	1.00	1.00	1.00
Junior high school	0.51 (0.92–0.91)	1.23 (0.65–2.30)	0.61 (0.45–0.83)	0.60 (0.42–0.84)	0.56 (0.29–1.08)	0.69 (0.34–1.37)
≥ Senior high school	0.23 (0.14–0.38)	2.59 (1.41–4.77)	0.30 (0.23–0.39)	0.48 (0.35–0.68)	0.20 (0.11–0.36)	0.69 (0.35–1.39)
Current smoker	3.80 (1.92–7.50)	1.21 (0.50–2.93)	2.23 (1.90–2.62)	1.44 (1.17–1.78)	1.54 (1.09–2.17)	0.80 (0.53–1.22)
Alcohol consumption	4.90 (2.28–10.51)	1.81 (0.71–4.63)	2.11 (1.80–2.48)	1.16 (0.94–1.44)	1.88 (1.30–2.74)	1.37 (0.86–2.18)
Diet rich in fruits and vegetables	1.99 (1.40–2.83)	1.42 (0.96–2.10)	1.36 (1.17–1.57)	1.27 (1.07–1.50)	0.95 (0.63–1.45)	0.72 (0.46–1.13)
High fat diet	0.51 (0.28–0.96)	0.60 (0.30–1.18)	0.64 (0.51–0.81)	0.75 (0.58–0.96)	0.62 (0.31–1.23)	1.02 (0.49–2.12)
Physical activity						
Low	1.00	1.00	1.00	1.00	1.00	1.00
Moderate	0.70 (0.47–1.04)	1.08 (0.69–1.68)	1.51 (1.30–1.76)	1.06 (0.89–1.27)	1.19 (0.77–1.86)	1.02 (0.63–1.66)
High	NA	NA	2.57 (1.71–3.86)	1.47 (0.93–2.30)	3.36 (1.42–7.96)	2.66 (1.05–6.72)
Body mass index						
Healthy weight	1.00	1.00	1.00	1.00	1.00	1.00
Underweight	0.54 (0.17–1.73)	1.26 (0.37–4.32)	0.61 (0.35–1.06)	0.91 (0.51–1.62)	0.46 (0.06–3.46)	0.95 (0.21–7.43)
Overweight	1.19 (0.80–1.77)	0.71 (0.45–1.11)	2.81 (2.35–3.37)	1.54 (1.26–1.88)	2.61 (1.53–4.46)	1.37 (0.79–2.38)
Obesity	1.75 (1.11–2.78)	1.05 (0.62–1.78)	4.99 (4.06–6.13)	2.30 (1.82–2.90)	4.90 (2.80–8.58)	2.47 (1.37–4.45)
Diabetes	2.94 (1.88–4.61)	1.13 (0.68–1.90)	5.06 (4.09–6.26)	2.71 (2.14–3.43)	NA	NA
Hypertension	2.90 (2.03–4.12)	1.81 (1.20–2.73)	4.61 (3.95–5.38)	2.79 (2.35–3.31)	NA	NA
Dyslipidemia	1.99 (1.33–2.97)	1.51 (0.96–2.36)	3.03 (2.54–3.60)	1.74 (1.43–2.12)	4.08 (2.67–6.23)	2.88 (1.84–4.51)
Hyperuricemia	1.39 (0.86–2.26)	1.57 (0.89–2.78)	1.70 (1.38–2.08)	0.96 (0.76–1.21)	1.16 (0.63–2.15)	0.65 (0.34–1.24)

Note: Data were crude and multivariable-adjusted odds ratio (95% Confidence Interval)

Abbreviation: *DKD* Diabetic kidney disease, *NA* Not applicable

Reference level: Gender = Woman; Education = Junior high school; alcohol consumption = No; diet rich in fruits and vegetables = No; high fat diet = No; physical activity = Low; body mass index = Healthy weight; Diabetes = No; Hypertension = No; Dyslipidemia = No; Hyperuricemia = No

In the ordinal logistic regression, the data were analyzed in two models (Table 7). In model one, age, smoking, a diet rich in fruits and vegetables, heavy physical activity, high BMI, diabetes, hypertension, and dyslipidemia were positively associated with increased severities of albuminuria in subjects with normal eGFR values, while a higher education level and a diet rich in meat were associated with reduced severities. Similarly, in model two, a higher education level and a high-fat diet were also negatively correlated with an elevated risk of renal damage, while older age, being a current smoker, a diet rich in fruits and vegetables, obesity, diabetes, hypertension and dyslipidemia showed a positive association.

**Table 7:** Results of ordinal logistic regression for two Models

	Model 1 (*N* = 5099)		Model 2 (*N* = 5231)	
Age	Adjusted OR	*P*	Adjusted OR	*P*
18-	1.00		1.00	
Age changed by 10 years	1.11 (1.02–1.21)	0.01	1.32 (1.22–1.43)	< 0.001
Gender				
Women	1.00		1.00	
Men	1.18 (0.95–1.47)	0.13	0.88 (0.72–1.08)	0.23
Education				
≤ Primary school	1.00		1.00	
Junior high school	0.60 (0.42–0.85)	0.004	0.68 (0.49–0.93)	0.02
≥ Senior high school	0.46 (0.32–0.65)	< 0.001	0.61 (0.45–0.84)	0.002
Current smoker	1.44 (1.17–1.79)	< 0.001	1.41 (1.15–1.74)	0.001
Alcohol consumption	1.15 (0.93–1.43)	0.20	1.10 (0.89–1.36)	0.39
Diet rich in fruits and vegetables	1.30 (1.10–1.54)	0.002	1.32 (1.13–1.55)	0.001
High fat diet	0.73 (0.56–0.95)	0.02	0.73 (0.57–0.93)	0.01
Physical activity				
Low	1.00		1.00	
Moderate	1.07 (0.89–1.28)	0.49	1.07 (0.90–1.27)	0.42
High	1.57 (1.01–2.44)	0.05	1.46 (0.94–2.27)	0.09
Body mass index				
Underweight	1.00		1.00	
Healthy weight	1.21 (0.66–2.20)	0.54	1.13 (0.66–1.95)	0.66
Overweight	1.82 (1.00–3.33)	0.05	1.52 (0.88–2.64)	0.13
Obesity	2.71 (1.47–5.00)	< 0.001	2.34 (1.34–4.11)	< 0.001
Diabetes	2.98 (2.35–3.77)	< 0.001	2.72 (2.18–3.40)	< 0.001
Hypertension	2.80 (2.35–3.34)	< 0.001	2.73 (2.32–3.22)	< 0.001
Dyslipidemia	1.81 (1.49–2.20)	< 0.001	1.79 (1.48–2.15)	< 0.001
Hyperuricemia	0.99 (0.78–1.25)	0.93	1.09 (0.87–1.36)	0.45

Note: Data were multivariable-adjusted odds ratio (95% Confidence Interval) and *P* value for each variable

Abbreviation: *NA* Not applicable

Reference level: Gender = Woman; Education = Primary school; current smoker = No; alcohol consumption = No; diet rich in fruits and vegetables = No; high fat diet = No; physical activity = Low; body mass index = Underweight; Diabetes = No; Hypertension = No; Dyslipidemia = No; Hyperuricemia = No.

## Discussion

According to the 2009 China Population Census, there are about 8% citizens in China living in Henan province. Zhengzhou, the capital city of Henan province, has a population of nearly 10 million and is one of the representative urban centers in Central China. To our knowledge, the current study is the first one, which performed with a large representative sample of an urban population in Central China, assessing the current epidemiological features of both vital indicators of renal damage, eGFR and albuminuria, and DKD. In our study, the overall prevalence of CKD and DKD was 16.8 and 3.5%, respectively, corresponding to over 3 million urban adults in Henan Province. Generally, older age, sex, education, smoking, unhealthy BMI, diabetes, hypertension, dyslipidemia and hyperuricemia were significantly associated with a higher risk for and elevated severities of reduced renal function. Compared with previous studies, these findings indicated that the prevalence of CKD was higher in the urban population in Central China than in the urban populations in South China (12.1%) and North China (13.0%) and that the prevalence of CKD had increased by 6% since 2009 (10.5%) [14, 28, 29].

In 2012, Zhang et al. reported that the prevalence of CKD in Chinese urban residents was 8.9%, with 2.3% of subjects exhibiting a reduced eGFR and 7.0% of subjects exhibiting albuminuria [30]. Our current study indicates that the number of people with CKD has increased in the past six years. Previous studies indicated that older age was an independent risk factor for reduced renal function, which was further supported by current study [14, 31–35]. Aging has been a serious social problem in China. The 2009 China Population Census indicated that residents living in Henan who aged over 50 and 60 years old contributed to 24.0 and 12.7% of total population, respectively. In current study, the mean age of the subjects without renal damage was 40.3 years, while the mean ages of those with reduced eGFR, albuminuria and DKD were 63.0, 51.5 and 57.9 years, respectively. The high prevalence of CKD partly attributed to this age distribution.

Diabetes and hypertension are reported to be significantly related to the high prevalence and incidence of CKD [6, 36–38]. In the past twenty years, a noteworthy increase in the prevalence of diabetes and hypertension in the Chinese population occurred. Xiang et al. conducted a national survey in China and demonstrated that the prevalence of impaired glucose tolerance and diabetes mellitus was 3.2 and 4.8%, respectively [39]. In 2004, the Fourth National Health and Nutrition Examination Survey of China (NHANES) reported that the prevalence of diabetes had increased to 6.4% [40]. Generally, the number of diabetic subjects in Chinese population has increased by 62.7% from 2000 to 2016. The GBD study estimated that there were almost 90 million Chinese people having diabetes in 2016 [41]. In 2002, Gu et al. suggested that the prevalence of hypertension was 13.6% in China [42]. The International Collaborative Study of Cardiovascular Disease in Asia further reported that there were 27.2% middle-age Chinese people having hypertension in 2012 [43]. Subsequently, a national survey reported that hypertension affected nearly 23.2% of Chinese adults in 2015 [44]. The changing trajectories of the prevalence of diabetes and hypertension were associated with CKD. In our study, we found that the majority of subjects with both hypertension and diabetes had reduced eGFR and albuminuria. The results of the logistic regression also showed that hypertension was independently associated with a higher risk of reduced eGFR, with an OR of 1.81, and both diabetes and hypertension were associated with a higher risk of albuminuria, with ORs of 2.71 and 2.79, respectively. Therefore, the high proportions of subjects with diabetes (7.6%) and hypertension (34.6%) could be partly contributed to the higher prevalence of CKD in our study population.

Previous studies suggest that dyslipidemia always develop along with kidney function decline in patients with CKD, even in the early stages. It is also the major risk factor for cardiovascular disease in CKD and ESRD patients [45]. Ji et al. found that increased serum concentrations of total cholesterol and triglycerides were significantly associated with mildly reduced eGFR [46]. Thompson et al. also indicated that decreased eGFR was independently associated with lower concentrations of high-density lipoprotein and higher concentrations of triglycerides in an Australian population [47]. In a longitudinal study, Tsai et al. found that the level of total cholesterol, both at baseline and over the longitudinal course, was significantly associated with a higher risk of incident ESRD [48]. Similarly, in our current study, the serum concentration of total cholesterol was much higher in subjects with reduced eGFR than in subjects without reduced eGFR, and subjects with DKD had the highest serum concentration of triglycerides. The results of both the binary and ordinal logistic regressions also indicated that dyslipidemia was significantly associated with albuminuria, DKD and severe renal damage.

Hyperuricemia generates renal injury via its crystal-independent mechanisms, such as activating the renin-angiotensin system, thereby inducing endothelial dysfunction and oxidative stress Elevated serum concentration of uric acid could activate the renin-angiotensin system and further induce endothelial dysfunction and oxidative stress which generates kidney injuries [49]. Previously, Weiner et al. observed that each 1 mg/dL increase in serum uric acid from baseline level was associated with a 7% higher risk of reduced eGFR in the Atherosclerosis Risks in Communities and the Cardiovascular Health Study [50]. In another cohort study, Zhang et al. reported that elevated concentration of serum uric acid was associated with new-onset albuminuria and reduced eGFR(defined as a decrease ≥20%) [51]. A meta-analysis integrating 13 cohort studies suggested that an elevated serum concentration of uric acid contribute to a 15% higher risk of new-onset CKD at follow-up and hyperuricemia was independently associated with a twofold higher risk of newly diagnosed CKD in subjects with normal kidney function at baseline [52].. In the current study, the prevalence of hyperuricemia was much higher in subjects with reduced eGFR and albuminuria than in subjects without renal damage (16.5 and 15.2%, respectively, versus 10.4%). Results of logistic regression also supported that hyperuricemia was significantly associated with a higher risk of albuminuria and DKD, with ORs of 1.70 and 2.15, respectively.

Logistic regression indicated that diet rich in fruits and vegetables was associated with a higher risk of albuminuria and the high fat diet had an opposite effect. These results were partly inconsistent with results of previous studies [53, 54]. A potential explanation for this phenomenon is that some of participants who consume more vegetables than meat because they can’t afford the Western-style diet. On the other hand, participants with high fat diet always have better social economic status. Similarly, heavy physical activities are also associated with engaging long labor hour job and relative lower social economic status.

Previously, we reported that the prevalence of CKD and DKD in Chinese rural population was 16.4 and 2.9% [55]. Comparing with it, our current study further demonstrated that the prevalence of CKD in Chinese urban population was similar to which we found in rural population, but the prevalence of DKD was much lower. Diabetes, hypertension and hyperuricemia were risk factors for both rural and urban population. But heavy physical activity was a risk factor for urban population rather than sedentary behavior for rural population. Standardized survey tools, trained medical practitioners and good sample size ensured the robustness of our results. However, several limitations should be addressed. Firstly, the serum creatinine and urinary albumin extraction were acquired from single measurements, which might partly overestimate the prevalences of CKD and DKD. Secondly, a cross-sectional study is incapable of demonstrating causal relationships between each risk factor and renal indicators. Thirdly, for subjects who were taking anti-hypertensive drugs, we did not specifically analyze the association between RAS blockers and DKD.

## Conclusion

The results of our current study demonstrate that the prevalences of CKD and DKD are still high in Chinese urban population and they are going to be a major social-economic burden in China. High prevalent hypertension and diabetes are primary risk factors for them. Specific strategies and interventions aimed at reducing the burden of CKD and DKD are urgently needed.

## Data Availability

The datasets used and/or analyzed during the current study are available from the corresponding author on reasonable request.

## Abbreviations

CKD: Chronic kidney disease
ESRD: End-stage renal disease
DKD: Diabetic kidney disease
BP: Blood pressure
BMI: Body mass index
SBP: Systolic blood pressure
DBP: Diastolic blood pressure
FPG: Fasting plasma glucose
ACR: Albumin to creatinine ratio
eGFR: Estimated glomerular filtration rate
KDIGO: Kidney disease: improving global outcomes
ADA: American diabetes association
IPAQ: International physical activity questionnaire
SD: Standard deviation
OR: Odds ratio
CI: Confidence interval
NHANES: National health and nutrition examination survey
DM: Diabetes mellitus
HTN: Hypertension

## References

[1] James SL, Abate D, Abate KH, Abay SM, Abbafati C, Abbasi N (2018). Global, regional, and national incidence, prevalence, and years lived with disability for 354 diseases and injuries for 195 countries and territories, 1990-2017: a systematic analysis for the global burden of disease study 2017. Lancet.

[2] Roth GA, Abate D, Abate KH, Abay SM, Abbafati C, Abbasi N (2018). Global, regional, and national age-sex-specific mortality for 282 causes of death in 195 countries and territories, 1980-2017: a systematic analysis for the global burden of disease study 2017. Lancet.

[3] Kyu HH, Abate D, Abate KH, Abay SM, Abbafati C, Abbasi N (2018). Global, regional, and national disability-adjusted life-years (DALYs) for 359 diseases and injuries and healthy life expectancy (HALE) for 195 countries and territories, 1990-2017: a systematic analysis for the global burden of disease study 2017. Lancet.

[4] Stanaway JD, Afshin A, Gakidou E, Lim SS, Abate D, Abate KH (2018). Global, regional, and national comparative risk assessment of 84 behavioural, environmental and occupational, and metabolic risks or clusters of risks for 195 countries and territories, 1990-2017: a systematic analysis for the global burden of disease study 2017. Lancet.

[5] Song K-K, Zhao D-L, Wang Y-D, Wang Y, Sun X-F, Miao L-N (2017). Analysis of factors associated with death in maintenance hemodialysis patients: a multicenter study in China. Chin Med J.

[6] Chen J (2010). Epidemiology of hypertension and chronic kidney disease in China. Curr Opin Nephrol Hypertens.

[7] Chung H-F, Al Mamun A, Huang M-C, Long KZ, Huang Y-F, Shin S-J (2017). Obesity, weight change, and chronic kidney disease in patients with type 2 diabetes mellitus: a longitudinal study in Taiwan. J Diabetes.

[8] Liu M, Liu SW, Wang LJ, Bai YM, Zeng XY, Guo HB (2019). Burden of diabetes, hyperglycaemia in China from to 2016: findings from the 1990 to 2016, global burden of disease study. Diabetes Metab.

[9] Zhang L, Zuo L, Wang F, Wang M, Wang S, Liu L (2007). Metabolic syndrome and chronic kidney disease in a Chinese population aged 40 years and older. Mayo Clin Proc.

[10] Yang W, Lu J, Weng J, Jia W, Ji L, Xiao J (2010). Prevalence of diabetes among men and women in China. N Engl J Med.

[11] Ho Y-W, Chau K-F, Choy B-Y, Fung K-S, Cheng Y-L, Kwan T-H (2010). Hong Kong renal registry report 2010. Hong Kong J Nephrol.

[12] Hwang SJ, Tsai JC, Chen HC (2010). Epidemiology, impact and preventive care of chronic kidney disease in Taiwan. Nephrology (Carlton).

[13] Wang J, Zhang L, Wang F, Liu L, Wang H (2014). Prevalence, awareness, treatment, and control of hypertension in China: results from a national survey. Am J Hypertens.

[14] Shan Y, Zhang Q, Liu Z, Hu X, Liu D (2010). Prevalence and risk factors associated with chronic kidney disease in adults over 40 years: a population study from Central China. Nephrology (Carlton).

[15] Liu X, Li Y, Guo Y, Li L, Yang K, Liu R (2018). The burden, management rates and influencing factors of high blood pressure in a Chinese rural population: the rural diabetes, obesity and lifestyle (RuralDiab) study. J Hum Hypertens.

[16] Liu X, Li Y, Li L, Zhang L, Ren Y, Zhou H (2016). Prevalence, awareness, treatment, control of type 2 diabetes mellitus and risk factors in Chinese rural population: the RuralDiab study. Sci Rep.

[17] Jha V (2010). Herbal medicines and chronic kidney disease. Nephrology (Carlton).

[18] Yang B, Xie Y, Guo M, Rosner MH, Yang H, Ronco C (2018). Nephrotoxicity and Chinese herbal medicine. Clin J Am Soc Nephrol.

[19] Garg AX, Kiberd BA, Clark WF, Haynes RB, Clase CM (2002). Albuminuria and renal insufficiency prevalence guides population screening: results from the NHANES III. Kidney Int.

[20] Ramirez SP, McClellan W, Port FK, Hsu SI (2002). Risk factors for proteinuria in a large, multiracial, southeast Asian population. J Am Soc Nephrol.

[21] Chen N, Wang W, Huang Y, Shen P, Pei D, Yu H (2009). Community-based study on CKD subjects and the associated risk factors. Nephrol Dial Transplant.

[22] Liu LS. [2010 Chinese guidelines for the management of hypertension]. Zhonghua Xin Xue Guan Bing Za Zhi 2011 Jul;39(7):579–615. 22088239

[23] Levey AS, Stevens LA, Schmid CH, Zhang YL, Castro AF, Feldman HI (2009). A new equation to estimate glomerular filtration rate. Ann Intern Med.

[24] Chapter 1: Definition and classification of CKD. Kidney Int Suppl. 2013;3(1):19–62. 10.1038/kisup.2012.64 PMC4089693 25018975

[25] [Chinese guidelines on prevention and treatment of dyslipidemia in adults]. Zhonghua Xin Xue Guan Bing Za Zhi. 2007 May;35(5):390–419. 17711682

[26] Wang S-S, Lay S, Yu H-N, Shen S-R (2016). Dietary guidelines for Chinese residents (2016): comments and comparisons. J Zhejiang Univ Sci B.

[27] Craig CL, Marshall AL, Sjostrom M, Bauman AE, Booth ML, Ainsworth BE (2003). International physical activity questionnaire: 12-country reliability and validity. Med Sci Sports Exerc.

[28] Zhang L, Zhang P, Wang F, Zuo L, Zhou Y, Shi Y (2008). Prevalence and factors associated with CKD: a population study from Beijing. Am J Kidney Dis.

[29] Chen W, Chen W, Wang H, Dong X, Liu Q, Mao H (2009). Prevalence and risk factors associated with chronic kidney disease in an adult population from southern China. Nephrol Dial Transplant.

[30] Zhang L, Wang F, Wang L, Wang W, Liu B, Liu J (2012). Prevalence of chronic kidney disease in China: a cross-sectional survey. Lancet..

[31] Liu ZH (2013). Nephrology in China. Nat Rev Nephrol.

[32] Chen J, Wildman RP, Gu D, Kusek JW, Spruill M, Reynolds K (2005). Prevalence of decreased kidney function in Chinese adults aged 35 to 74 years. Kidney Int.

[33] Zhang L, Zuo L, Xu G, Wang F, Wang M, Wang S (2007). Community-based screening for chronic kidney disease among populations older than 40 years in Beijing. Nephrol Dial Transplant.

[34] Xie Y, Chen X (2008). Epidemiology, major outcomes, risk factors, prevention and management of chronic kidney disease in China. Am J Nephrol.

[35] Chen W, Liu Q, Wang H, Chen W, Johnson RJ, Dong X (2011). Prevalence and risk factors of chronic kidney disease: a population study in the Tibetan population. Nephrol Dial Transplant.

[36] Cao Y, Li W, Yang G, Liu Y, Li X (2012). Diabetes and hypertension have become leading causes of CKD in Chinese elderly patients: a comparison between 1990-1991 and 2009-2010. Int Urol Nephrol.

[37] Qin X, Wang Y, Li Y, Xie D, Tang G, Wang B (2015). Risk factors for renal function decline in adults with normal kidney function: a 7-year cohort study. J Epidemiol Community Health.

[38] Levey AS, Bilous R, Shlipak MG (2016). CKD and diabetes: what can we learn from their similarities and differences?. Am J Kidney Dis.

[39] Hongding X, Wei W, Canqun L (1998). A epidemiological study on DM 1995-1996 in China. Chin J Diab.

[40] Danaei G, Finucane MM, Lu Y, Singh GM, Cowan MJ, Paciorek CJ (2011). National, regional, and global trends in fasting plasma glucose and diabetes prevalence since 1980: systematic analysis of health examination surveys and epidemiological studies with 370 country-years and 2· 7 million participants. Lancet..

[41] Liu M, Liu SW, Wang LJ, Bai YM, Zeng XY, Guo HB (2019). Burden of diabetes, hyperglycaemia in China from to 2016: Findings from the 1990 to 2016, global burden of disease study. Diabetes Metabol.

[42] Tao S, Wu X, Duan X, Fang W, Hao J, Fan D (1995). Hypertension prevalence and status of awareness, treatment and control in China. Chin Med J (Engl)..

[43] Gu D, Reynolds K, Wu X, Chen J, Duan X, Muntner P (2002). Prevalence, awareness, treatment, and control of hypertension in China. Hypertension..

[44] Wang Z, Chen Z, Zhang L, Wang X, Hao G, Zhang Z (2018). Status of hypertension in China: results from the China hypertension survey, 2012-2015. Circulation.

[45] Hager MR, Narla AD, Tannock LR (2017). Dyslipidemia in patients with chronic kidney disease. Rev Endocr Metab Disord.

[46] Ji B, Zhang S, Gong L, Wang Z, Ren W, Li Q, et al. The risk factors of mild decline in estimated glomerular filtration rate in a community-based population. Clin Biochem. 2013;46(9):750–4.43. Michael Thompson, Udayan Ray, Richard Yu, Andrew Hudspeth, Michael Smillie, Neville Jordan, et al. Kidney function as a determinant of HDL and triglyceride concentrations in the Australian population. J Clin Med. 2016;5(3):35. 10.3390/jcm5030035 PMC4810106 27005668

[47] Thompson M, Ray U, Yu R, Hudspeth A, Smillie M, Jordan N (2016). Kidney function as a determinant of HDL and triglyceride concentrations in the Australian population. J Clin Med.

[48] Tsai CW, Huang HC, Chiang HY, Chung CW, Chang SN, Chu PL (2019). Longitudinal lipid trends and adverse outcomes in patients with CKD: a 13-year observational cohort study. J Lipid Res.

[49] Isaka Y, Takabatake Y, Takahashi A, Saitoh T, Yoshimori T (2016). Hyperuricemia-induced inflammasome and kidney diseases. Nephrol Dial Transplant.

[50] Weiner DE, Tighiouart H, Elsayed EF, Griffith JL, Salem DN, Levey AS (2008). Uric acid and incident kidney disease in the community. J Am Soc Nephrol.

[51] Zhang L, Wang F, Wang X, Liu L, Wang H (2012). The association between plasma uric acid and renal function decline in a Chinese population-based cohort. Nephrol Dial Transplant.

[52] Li L, Yang C, Zhao Y, Zeng X, Liu F, Fu P (2014). Is hyperuricemia an independent risk factor for new-onset chronic kidney disease?: a systematic review and meta-analysis based on observational cohort studies. BMC Nephrol.

[53] Odermatt A (2011). The Western-style diet: a major risk factor for impaired kidney function and chronic kidney disease. Am J Physiol Renal Physiol.

[54] Friedman AN (2004). High-protein diets: potential effects on the kidney in renal health and disease. Am J Kidney Dis: Official J Natl Kidney Found.

[55] Duan J, Wang C, Liu D, Qiao Y, Pan S, Jiang D (2019). Prevalence and risk factors of chronic kidney disease and diabetic kidney disease in Chinese rural residents: a cross-sectional survey. Sci Rep.

